# Spatial Analysis of County-Level Breast Cancer Mortality in Texas

**DOI:** 10.1155/2012/959343

**Published:** 2012-01-31

**Authors:** Arvind B. Bambhroliya, Keith D. Burau, Ken Sexton

**Affiliations:** ^1^Division of Epidemiology, Human Genetics and Environmental Science, University of Texas School of Public Health, 1200 Herman Pressler, Houston, TX 77030, USA; ^2^Division of Biostatistics, University of Texas School of Public Health, 1200 Herman Pressler, Houston, TX 77030, USA; ^3^Division of Epidemiology, Human Genetics and Environmental Science, University of Texas School of Public Health, Brownsville Regional Campus, 80 Fort Brown Road, RAHC, Brownsville, TX 78520, USA

## Abstract

*Objective*. The objectives of the study were to detect high-risk areas and to examine how racial and ethnic status affect the geographic distribution of female breast cancer mortality in Texas. Analyses were based on county-level data for the years from 2000 to 2008. *Materials and Methods*. Breast cancer mortality data were obtained from the Texas Cancer Registry, and the Spatial Scan Statistics method was used to run Purely Spatial Analyses using the Discrete Poisson, Bernoulli, and Multinomial models. *Results and Conclusions*. Highest rates of female breast cancer mortality in Texas have shifted over time from southeastern areas towards northern and eastern areas, and breast cancer mortality at the county level is distributed heterogeneously based on racial/ethnic status. Non-Hispanic blacks were at highest risk in the northeastern region and lowest risk in the southern region, while Hispanics were at highest risk in the southern region along the border with Mexico and lowest risk in the northeastern region.

## 1. Introduction

Breast cancer is the most common cancer and second leading cause of cancer mortality in the United States and Texas [1]. It is estimated that 207,090 new cases of invasive breast cancer and 39,840 breast cancer deaths occurred among women in the USA during 2010 [1]. There is considerable variation in the rates of breast cancer mortality at the county level. The reported highest and lowest age-adjusted mortality rates for breast cancer from 2000 to 2008 for Texas at the county level were 41.9 and 16.1 per 100,000, respectively [2]. Apart from geographical variation in breast cancer mortality, there is a significant disparity in breast cancer mortality in Texas based on racial and ethnic status. Mortality rates for female breast cancer were approximately 30% higher during 1992–2001 [3] and 50% higher during 2001–2005 [4] among black women compared to white women. Rates were lower among Hispanic compared to white women [3, 4]. Both black and Hispanic women were found to have significantly increased relative risk of breast cancer mortality compared to non-Hispanic white women during 1992–2000 [3, 5].

Relatively few studies have been conducted to examine how racial and ethnic status affects the geographic distribution of breast cancer mortality, and no study has been conducted to investigate this at the county level in the state of Texas. Identification of geographic patterns of breast cancer mortality based on racial and ethnic status could provide impetus to conduct further investigations and target health resources for prevention and treatment in specific geographic areas. The Spatial Scan Statistic method developed by Kulldorff [6], Kulldorff and Nagarwalla [7] has been shown to be an effective method for investigating geographical patterns of cancer and detecting spatial cancer clusters. A cancer cluster is defined as a greater-than-expected number of cancer cases that occurs within a group of people in a geographic area over a specified period of time. 

A previous study published by Zhan and Lin in 2003 [8] detected two statistically significant clusters of female breast cancer mortality at the county level using data provided by the Bureau of Vital Statistics of the Texas Department of Health for the period from 1990 to 1997. The most likely cluster identified (*P* value 0.0004) was located in the southeastern portion of Texas, including these 38 counties—Aransas, Atascosa, Austin, Bastrop, Bee, Bexar, Brazoria, Brooks, Caldwell, Calhoun, Colorado, De Witt, Duval, Fayette, Fort Bend, Galveston, Goliad, Gonzales, Guadalupe, Harris, Jackson, Jim Hogg, Jim Wells, Karnes, Kenedy, Kleberg, La Salle, Lavaca, Live Oak, Matagorda, McMullen, Nueces, Refugio, San Patricio, Victoria, Waller, Wharton, Willacy, and Wilson. A secondary cluster (*P* value 0.0077) included only one county: El Paso. In this paper, we present results of spatial analyses using the Spatial Scan Statistic method on female breast cancer in the state of Texas using county-level female breast cancer mortality data for the years 2000 to 2008. The objectives of the study were to detect high-risk areas of breast cancer mortality through the Discrete Poisson model, investigate whether there are high-risk areas where the distribution of breast cancer mortality differs based on the racial/ethnic status of the population using the Multinomial model, and examine the spatial distribution of breast cancer mortality cases for non-Hispanic blacks (NHBs), Hispanics and other races compared to non-Hispanic whites (NHWs) using the Bernoulli model.

## 2. Materials and Methods

### 2.1. Geographic, Population, and Mortality Data

The case definition for this study was a death due to malignant neoplasm of breast cancer (C50) as listed by the International Classification of Diseases, 10th Revision (ICD-10) in the female population of Texas for the years 2000 to 2008. The breast cancer mortality data at the county level for the years 2000 to 2008 were obtained from the Texas Cancer Registry, Cancer Epidemiology and Surveillance Branch, Texas Department of State Health Services. The Texas Cancer Registry classifies cancer mortality data according to the Surveillance, Epidemiology, and End Results (SEER) “Cause of Death Recode”, as given by the SEER Cause of Death Recode 1969+ (9/17/2004) (http://seer.cancer.gov/codrecode/1969+_d09172004/index.html), and the SEER program has defined major site groups based on the ICD-10. The Texas Cancer Registry is a statewide, population-based registry that collects high-quality, population-based data reported from various sources, including hospitals, cancer treatment centers, ambulatory surgery centers, pathology laboratories, and physician's offices through active and passive surveillance. It currently meets standards set by the National Program of Central Cancer Registries, Centers for Disease Control and Prevention for high-quality data, and is Gold-Certified by the North American Association of Central Cancer Registries.

In all, 22,820 breast cancer deaths occurred in Texas between the years 2000 to 2008, of which 15,234 (66.8%) were attributed to NHW, 3,503 (15.4%) to NHB, and 3,770 (16.5%) to Hispanics. At-risk population data at the county level for Texas covering the same time period were obtained from the US Census Bureau. Geographic coordinate data (i.e., county centroids) that represent the locations of the 254 Texas county polygons were specified by the 2000 US Bureau of the Census.

### 2.2. Statistical Analyses

We used Spatial Scan Statistics to examine the presence of breast cancer clusters. We ran Purely Spatial Analyses using 4 different models (Discrete Poisson with and without covariates, Bernoulli and Multinomial models). For the Discrete Poisson model, we assumed that the number of deaths in each county was Poisson distributed and ran the model with and without adjustment of racial/ethnic status of the population. For the Bernoulli model, we classified breast cancer deaths among NHW as controls and breast cancer deaths among other racial/ethnic groups as cases, thereby creating 3 comparisons (NHB versus NHW, Hispanics versus NHW and “other races” versus NHW). For the Multinomial model, we classified breast cancer deaths into 4 racial/ethnic categories (NHW, NHB, Hispanics, and other races). The level of statistical significance used for this study was 0.05. We used the SaTScan v9.1.1 released on March 9, 2011 to run the Spatial Scan Statistics. The Spatial Scan Statistics detect high-risk areas of cases by gradually scanning a window across space and noting the number of observed and expected observations inside the window. The window sizes are varied continuously up to a prespecified maximum size. The most likely cluster (the cluster least likely to be due to chance) is assigned to a window with the maximum likelihood. We used the scanning window in the shape of a circle and specified the maximum window size as one that included 50% of the at-risk population throughout our analyses. We also report secondary clusters that cause a rejection of the null hypothesis (i.e., the log likelihood ratio of secondary clusters in the real data is higher than that of the most likely cluster in the simulated data sets) and do not overlap the most likely cluster. MapInfo Professional version 8.0 was used to create the thematic map based on detected counties by Spatial Scan Statistics.

### 2.3. Study Approval

This study did not need to be approved by the Committee for the Protection of Human Subjects because it used aggregated data on county-level breast cancer mortality in Texas.

## 3. Results and Discussion

### 3.1. Results

#### 3.1.1. Breast Cancer Clusters Using the Discrete Poisson Model

**Figure 1:**
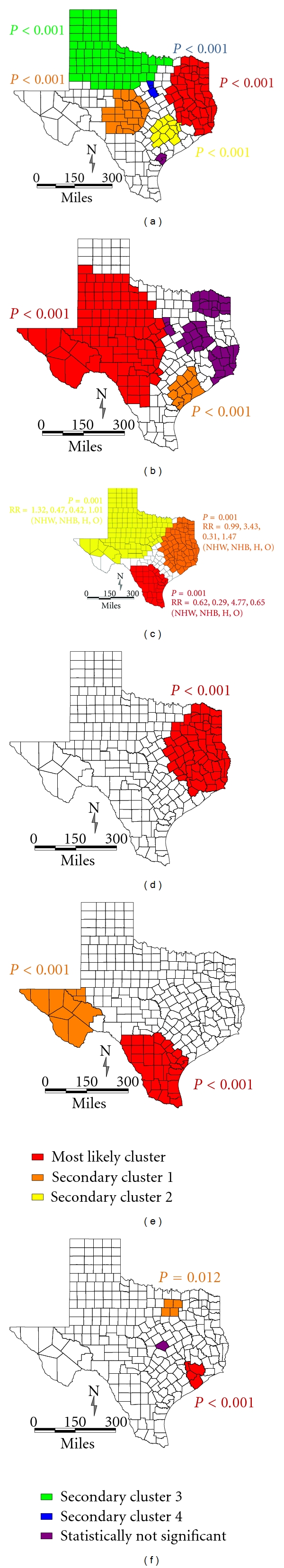
Breast cancer mortality clusters at the county level in Texas for the years 2000 to 2008 using Discrete Poisson model without covariate adjustment (a), Discrete Poisson model with race/ethnicity as covariate (b), Multinomial model (c), Bernoulli model—non-Hispanic blacks (NHBs) versus non-Hispanic whites (NHWs) (d), Bernoulli model—Hispanics (H) versus NHW (e) and Bernoulli model: other races (O) versus NHW (f).

Results of spatial analysis using the Discrete Poisson model without any covariate adjustment suggests that there were five statistically significant clusters of counties with a high rate of female breast cancer mortality in Texas for the years 2000 to 2008 (Table 1(a), Figure 1(a)). The most likely cluster was located in the northeast corner of Texas, including 51 counties with relative risk of 1.27 compared to the rest of Texas. Secondary clusters were located in the central region of Texas; the southeast central region; the northern part of the state; the region inclusive of Bosque, Hood and Somervell counties. Corresponding relative risks were 1.57, 1.38, 1.13, and 1.66, respectively, compared to the rest of Texas. After adjusting for racial/ethnic status of the population, spatial analysis using the Discrete Poisson model detected two statistically significant clusters (Table 1(b), Figure 1(b)). The most likely cluster was located in the western and central region of Texas, including 106 counties with relative risk of 1.23 compared to the rest of the state. A secondary cluster was located in southeastern Texas with relative risk of 1.32 compared to the rest of Texas.

#### 3.1.2. Breast Cancer Clusters Using the Multinomial Model

Results of spatial analysis using the Multinomial model suggest that there were three statistically significant clusters of counties where the distribution of risk for female breast cancer mortality based on race/ethnicity is statistically significantly different from the remaining regions of Texas (Table 2, Figure 1(c)). The most likely cluster was located in south Texas where Hispanics had the highest risk and NHB had the lowest risk. The risk of female breast cancer mortality was 4.77 times higher among Hispanics and 0.29 times lower among NHB in the cluster compared to the rest of Texas. The first secondary cluster was located in northeast Texas where NHB had the highest risk and Hispanics had the lowest. The risk of female breast cancer mortality was 3.42 times higher among NHB and 0.31 times lower among Hispanics in this cluster compared to the rest of Texas. The other secondary cluster was located in the northern and western region of Texas, where NHW had the highest risk and Hispanics had the lowest, although the risk of female breast cancer mortality for one group is not substantially different from the other groups.

#### 3.1.3. Breast Cancer Clusters Using the Bernoulli Model

Table 3 shows results of spatial analysis using the Bernoulli model for NHB, Hispanics, and other races compared to NHW. For NHB versus NHW, we detected one significant cluster located in the northeastern region of Texas, with relative risk of 2.63 compared to the rest of the state (Figure 1(d)). For Hispanics versus NHW, we detected two significant clusters (Figure 1(e)). The most likely cluster was located in the extreme southern region of Texas with relative risk of 4.13 compared to the rest of Texas, and the secondary cluster was located in the western region of Texas with relative risk of 3.83 compared to the rest of the state. For “other races” versus NHW, we detected two significant clusters (Figure 1(e)). The most likely cluster included four counties (Brazoria, Fort Bend, Harris, and Waller) in southeast Texas, with relative risk of 2.77, and the secondary cluster included four counties (Collin, Dallas, Denton, and Tarrant) in northeast Texas, with relative risk of 1.70 compared to the rest of the state.

### 3.2. Discussion

We found several statistically significant clusters for female breast cancer mortality at the county level in Texas through Purely Spatial Analyses. Five significant clusters were found through the Discrete Poisson model without any covariate adjustment, while the same model after adjusting for racial/ethnic status detected two significant clusters with different geographic distributions. The Multinomial model detected three significant clusters with different distributions of risk based on racial/ethnic status. The Bernoulli model found one significant cluster for NHB versus NHW, while two significant clusters were detected for Hispanics versus NHW and another two for “other races” versus NHW.

Zhan and Lin (2003) conducted spatial cluster analysis using the Discrete Poisson model without any covariate adjustment on female breast cancer mortality data for the years 1990 to 1997 and found two significant clusters [8]. The most likely cluster was located in the southeast region of Texas and the secondary cluster included only one county: El Paso. We found five significant clusters using the same analysis for the years 2000 to 2008. The most likely cluster in our findings was located in the northeast region of Texas, and three secondary clusters were located in the northern and central regions of the state. Only one secondary cluster located in southeast Texas had overlapped with the most likely cluster found by Zhan and Lin (2003). This indicates that the geographic distribution of female breast cancer mortality at the county level has shifted over time from the southeast towards northern and eastern areas in Texas. One explanation for this change could be rapid growth of urban areas and slower growth of rural areas in Texas during the same period. There were significant population changes over the years 2000–2010 in Texas. In fact, Texas is one of the top five fastest growing states with a 20.6% and 4.3 million (highest numeric increase) population increase between 2000 and 2010 [9]. About 85% of the total Texas population resided in urban areas in 2005, with most concentrated in the six largest metropolitan statistical areas—Austin-San Marcos, Dallas, Fort Worth, El Paso, Houston, and San Antonio [10]. Houston and Dallas-Fort Worth were among the top 3 fastest growing metro areas of the ten most populous metro areas in the Unites States. Together, they accounted for almost one-half of the state population and its population growth between 2000 and 2010 [9]. Hispanics are the fastest growing population in Texas and are expected to outnumber Whites by 2020 [10]. This shift in population demographics presents challenges for effective distribution of health care resources to reduce the disparities for breast cancer mortality. After adjusting for racial and ethnic status, the location of the most likely cluster changed from the northeast to the western area of Texas. This indicates that the existence of clusters in northeast Texas could be explained by racial and ethnic disparities in the region, while other etiological factors might explain mortality clusters in west Texas.

We found three significant clusters using Multinomial analysis where risk for breast cancer mortality differed based on racial/ethnic status. The cluster in the northeast area of Texas had significantly highest risk for NHB and lowest risk of Hispanics, while the cluster in the southern area of Texas had significantly highest risk for Hispanics and lowest risk for NHB. It is important to note that risk is not substantially different among racial/ethnic groups in the northern Texas cluster. Findings from the Bernoulli model analysis support the results of the Multinomial analysis, indicating that breast cancer mortality is heterogeneously distributed based on racial/ethnic status. 

NHB and Hispanics are more likely to be diagnosed with later stages of breast cancer [11–13] and have relatively higher risk of breast cancer mortality compared to NHW [3, 5]. Socioeconomic status (SES) of the population is an important predictor of stage of breast cancer at diagnosis [14]. Less utilization of mammography facilities, cancer radiotherapy, and cancer surgery among minorities is the likely reason that they tend to be diagnosed at later stages and have higher mortality from breast cancer [15–17]. Hispanics were the majority in 34 Texas counties in the USA-Mexico border region [18], and health care, including preventive and therapeutic resources, are more likely to be scarce or lacking in this underserved population [19, 20]. All of these factors could help explain why the highest risks for NHB were in the northeast, and highest risks for Hispanics were in the southern areas of Texas. The lower risks for other racial/ethnic groups are the result of unknown factors.

We identified one cluster in northeast Texas that had higher risk of breast cancer mortality for NHB compared to NHW though the Bernoulli model analysis. The location of this cluster was the same as that found by the Multinomial analysis; however, the risk was slightly lower. This could be due to fact that we included other races in the Multinomial model analysis and breast cancer incidence and mortality have been observed to be lower among this group compared to NHW, NHB, and Hispanics. For example, cancer incidence and mortality per 100,000 were 51.7 and 8.6, respectively, among Asian/Pacific Islanders for the years 2001 to 2005, while these same rates were 125.4 and 24.3 among NHW, 116.2 and 35.6 among NHB, and 84.6 and 17.2 among Hispanics [21].

Using Bernouli model analysis, we identified two clusters, one in the western part of the state and another along the border with Mexico, where Hispanics had significantly higher risk of breast cancer mortality compared to NHW. The most likely cluster was the same as that found by the Multinomial analysis; however, the risk was slightly lower. For “other races,” two significant clusters were detected, with each covering four counties around the Houston and Dallas metropolitan areas, respectively. The cluster around Houston had higher risk compared to the Dallas area. This might be related to the fact that the proportion of total population for other races in Houston was higher compared to Dallas.

This type of cluster analysis at the county level can provide useful information to policy makers for the following reasons.

**Figure 2:**
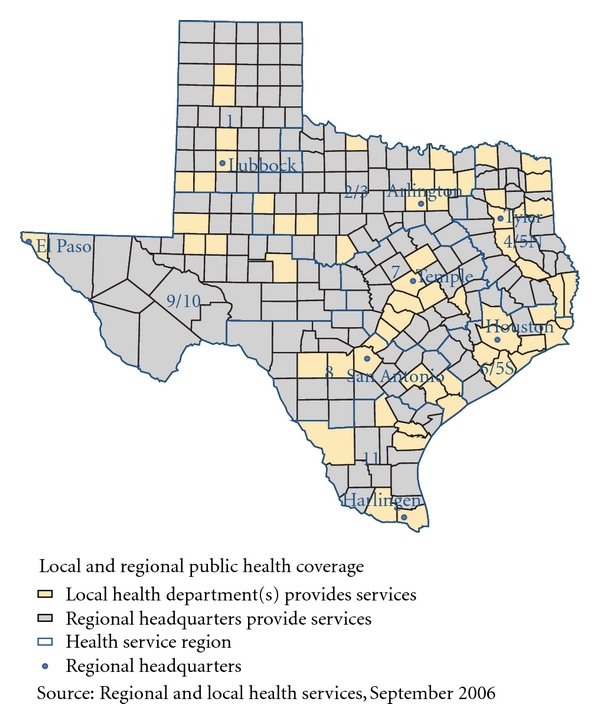
Map of Health Service Regions of the Department of State Health Services in Texas.

**Figure 3:**
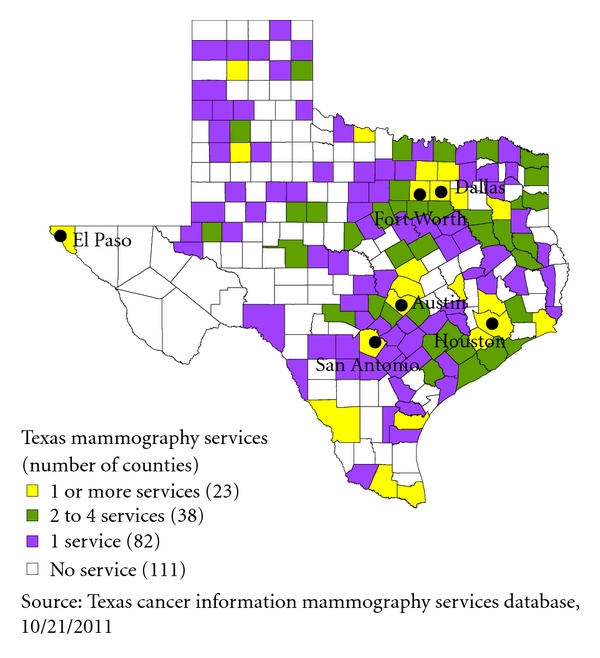
Map of accredited on-site or mobile mammography services by county.

1. The Department of State Health Services divides all Texas counties into Health Service Regions (HSRs), identified numerically from 1 to 11, to provide comprehensive public health services to the citizens of Texas through 8 regional public health offices (Figure 2) [22]. In addition, public health services in Texas are also provided through local health departments. But only one-fourth of counties in Texas have local health departments and most of them are located in urban areas. Results of our study could be important to regional public health offices and local health departments for establishing priorities and making strategic decisions about distribution of services for breast cancer. For example, secondary cancer cluster 2 identified through the Multinomial model (Figure 1(c)) covers HSRs 1, 2/3 and 9/10 and very few counties in these regions are covered through local health departments (Figure 2). Regional offices could utilize their limited resources to provide screening services for breast cancer in areas not covered by local health departments. Moreover, our results also indicate racial disparities in cluster identification that might be useful to regional and local health departments. For example, the most likely cluster identified through the Multinomial model (Figure 1(c)) had highest risk for Hispanics. Regional and local health departments could utilize this information to direct their resources for screening and education to this population.
2. Approximately half of Texas counties did not have accredited permanent mammography facilities in 2008 as reported in the Texas Cancer Facts & Figures, 2008 [21]. Moreover, most of counties with more than 1 accredited facility are located around the largest metro areas (Figure 3) [23]. Though the majority of the Texas population is concentrated in these areas, our findings provide important information that will help to target underserved populations in nonmetro areas. For example, on-site or mobile mammography facilities with more multilingual and follow-up supports could be introduced in the southern areas of Texas along the border with Mexico, where we identified a cancer cluster with significantly higher risk of breast cancer mortality among Hispanics compared to NHW (Figure 1(e)).
3. Results of our spatial analyses at the county level provide useful information to guide future spatial analyses at finer scales in Texas. Moreover, they are useful jumping-off points to conduct subcluster analyses at the county level or finer scales for a particular population group. For example, the census block or tract level analysis of female breast cancer mortality among NHB could be conducted in the HSRs 4, 5, 6, and 7 covering secondary cluster 2 identified through the Multinomial model (Figure 1(c)) to identify high risk areas for that particular population.

There are several limitations to the present study. We used breast cancer mortality data aggregated at the county level, which could affect the sensitivity of cluster detection. The geographic distribution of total population and number of breast cancer mortality cases at the county level in Texas is heterogeneous, with some counties having much lower mortality than other counties. This could affect study power so that some potential clusters might be missed. Lack of significance for some secondary clusters might be due to this reason or it might be due to the fact that the test is conservative, that is, we compared secondary clusters with the most likely cluster from the simulated datasets. Results may not be comparative to other studies using data aggregated at different geographic scales. Also, we included only race/ethnicity as a confounder in our analyses, yet there are many other known or hypothesized risk factors for breast cancer that we did not analyze, such as age at diagnosis [24], family history [25], alcohol consumption [26], access to mammography and other health care facilities [27, 28] and various environmental [29, 30] and reproductive factors [31, 32]. Inclusion of these factors might help to explain the existence of detected clusters. 

## 4. Conclusions

Results of our analyses indicate that breast cancer mortality at the county level in Texas is distributed heterogeneously based on racial/ethnic status. The evidence suggests that highest rates of female breast cancer mortality have shifted over time from southeastern areas towards northern and eastern areas of the state. In Texas, NHB had highest risk for breast cancer mortality in the northeastern region and lowest risk in the southern region, while Hispanics had highest risk in the southern region along the border with Mexico and lowest risk in the northeastern region. These findings, along with continuing trends toward urbanization, growing numbers of Hispanic residents, and increasing levels of poverty for many minorities, provide challenges and opportunities for Texas policy makers and health advocates. More research is needed to make informed decisions about effective and efficient distribution of health care resources to reduce breast cancer disparities for Texas residents.

## Figures and Tables

**Table 1:** List of breast cancer mortality clusters using Discrete Poisson model at county level in Texas for the years 2000 to 2008.

Cluster	Observed cases	Expected Cases	RR	LLR	*P *value
(a) Without covariate adjustment					
Most likely cluster	3388	2754.18	1.27	78.04	<0.001
Secondary cluster 1	496	319.35	1.57	42.43	<0.001
Secondary cluster 3	525	382.84	1.38	24.08	<0.001
Secondary cluster 4	1992	1775.27	1.13	13.84	<0.001
Secondary cluster 5	119	71.95	1.66	12.88	<0.001
Not significant cluster	52	33.23	1.57	4.52	0.654

(b) With adjustment of race/ethnic status of population					
Most likely cluster	4550	3843.34	1.23	74.66	<0.001
Secondary cluster 1	476	363.25	1.32	16.21	<0.001
Not significant cluster	119	85.39	1.40	5.91	0.26
Not significant cluster	1209	1111.12	1.09	4.41	0.68
Not significant cluster	713	640.85	1.12	4.04	0.80
Not significant cluster	413	370.87	1.12	2.35	1.00

Abbreviations: RR, Relative risk; LLR, Log-likelihood ratio.

**Table 2:** List of breast cancer mortality clusters using Multinomial model at county level in Texas for the years 2000 to 2008.

Cluster	Observed cases (NHW, NHB, H, O)	Expected cases (NHW, NHB, H, O)	RR (NHW, NHB, H, O)	LLR	*P *value
Most likely cluster	1437, 164, 1659, 32	2202.32, 505.41, 537.33, 46.94	0.62, 0.29, 4.77, 0.65	1327.21	0.001
Secondary cluster 1	7586, 2706, 887, 193	7607.78, 1745.89, 1856.16, 162.16	0.99, 3.43, 0.31, 1.47	1093.82	0.001
Secondary cluster 2	4348, 437, 419, 76	3532.28, 810.61, 861.81, 75.29	1.32, 0.47, 0.42, 1.01	418.51	0.001

Abbreviations: RR: relative risk; LLR: log-likelihood ratio; NHW: non-Hispanic whites; NHB: non-Hispanic blacks; H: Hispanics; O: other races.

**Table 3:** List of breast cancer mortality clusters for non-Hispanic blacks, Hispanics, and other races using Bernoulli model compared to non-Hispanic whites at county level in Texas for the years 2000 to 2008.

Cluster	Observed cases	Expected Cases	RR	LLR	*P *value
(a) Non-Hispanic blacks versus non-Hispanic whites					
Most likely cluster	2531	1744.27	2.63	447.78	<0.001

(b) Hispanics versus non-Hispanic whites					
Most likely cluster	1659	607.22	4.13	1122.37	<0.001
Secondary cluster	510	148.27	3.83	430.95	<0.001

(c) Other races versus non-Hispanic whites					
Most likely cluster	115	53.66	2.77	34.75	<0.001
Secondary cluster	101	68.04	1.70	9.36	0.012
Not significant cluster	10	3.90	2.61	3.47	0.89

Abbreviations: RR: relative risk; LLR: log-likelihood ratio.
